# Some Points in the Correction of Irregularities

**Published:** 1903-08-15

**Authors:** Russell Markwell


### SOME POINTS IN THE CORRECTION OF IRREGULARITIES.

BY DR. RUSSELL MARKWELL.

Time to Regulate.—It seems to be the opinion of a great many men in the profession that it is necessary to wait until all the permanent teeth are erupted before attempting to benefit a case of irregularity; but it is the universal opinion of the best authorities that the proper time is as soon as the teeth are erupted far enough to get bands on them.

Many a case of malocclusion could have been prevented by possibly removing one tooth at the proper time. We know if one tooth erupts out of line, it has its effect on the entire set. I do not desire to leave the impression that teeth can only be regulated while the patient is young, for age does not make a great deal of difference, so far as moving the teeth is concerned; but if we take them early in life, when the jaws are developing, the results are more satisfactory.

The first question that arises when a case of irregularity presents itself is, how shall we proceed?

Taking Impressions.—Take an impression and run models of both upper and lower. Do not take your impressions with modeling compound or wax, but take them in plaster. It may take a little longer, but you are sure of a perfect impression and it is the only way you can get one. Take pains in making your models, for they are of inestimable value to you. When you have perfect models, you can then study the occlusion from the lingual as well as the buccal side, and be able to get a more intelligent idea of the case than by studying the patient alone. Do not be too hasty in rendering an opinion, for you may have cause afterward to regret it; but be sure of what your results are going to be first.

Causes.—One of the first things we try to determine, after looking over a case, is the cause. I dare say the majority present is familiar with the principal causes, and it would be useless for one to enumerate them; but there is one point along this line I wish to call your attention, and that is regarding heredity. A great many seem to think it plays a very important part, but it is the opinion of men who have devoted their time to the study of this branch, that it has a very slight effect, and such cases as were formerly attributed to heredity are now largely attributed to degeneracy and lack of development.

One of the first and most important things to consider in studying a case is the occlusion.

Occlusion.—Occlusion is the fundamental principle governing the correction of irregularities. It is impossible to handle cases of orthodontia without a thorough knowledge of occlusion; when we are masters of this subject, regulating teeth will be as easy as any other branch in the profession.

A great many endeavor to correct irregularities without having any idea about occlusion; they seem to think that alignment of the teeth is all that is necessary.

It is not necessary to sacrifice facial expression to obtain normal occlusion. The two go hand-in-hand, and, as a rule, with a very rare exception, now and then, if we are able to correct the malocclusion, we need have no fear about the facial expression.

Many men will spend hours working on an artificial set of teeth, to get the articulation perfect, but how many seem to care to spend any time in trying to benefit a case of malocclusion? The sooner that every man in the profession realizes that occlusion comes before alignment in the correction of irregularities, that much sooner will he meet with success in the practice of orthodontia and desist from the almost universal practice of extracting.

Extracting.—It has been wisely said that, "every tooth is a key to the arch, and should be retained if possible." Nature has seen fit to provide us with a certain number of teeth, and man should not try to alter her laws, but endeavor to assist her.

It is not the mission of the orthodontist to combat nature in the development of the jaw by extracting teeth, but should be to do all in his power to overcome the degeneracy of mankind, which seems to be more pronounced with each generation.

Extracting is only permissible in very rare instances, and those cases are when the expansion would destroy the facial contour. The idea to extract to obtain room is absurd, for room is easily enough obtained with the proper appliances.

Appliances.—It would not be reasonable to expect every one to use the same kind of appliances. We find a number of different makes on the market, but, in looking them over, it can readily be seen that all the sets of appliances and methods of treatment of cases are focused toward one general method, with a little individuality as to detail.

Experience teaches me that it is not only better, but cheaper, to buy appliances than to make them.

Simplicity is next to efficiency in the application of regulating appliances.

There are a few points which I desire to call particular attention to regarding the application of appliances, and they are as to the use of the expansion arch, clamp bands, spur wire, band metal and metal ligature. With the judicious use of these, at least ninety-five percent of the cases can be handled successfully.

In selecting an expansion arch it is necessary, to take into consideration the age of the patient and the firmness of the teeth. If the patient is young, a lighter wire should be used than in a case that is well advanced.

The clamp band we can not get along without, and it is necessary that it be durable, simple and easy of application, for it is the clamp band that is destined to receive all the force used in moving the teeth. The band metal supplied by the manufacturers is all we would wish it to be, thin, easy of manipulation, and strong enough to withstand all the force we desire to use.

The metal ligature, which is annealed brass wire, ranging from guage 25 to 29, is recommended to the almost exclusion of any other kind of ligature, by the best posted men in orthodontia. With the use of the metal ligature, we are able to eliminate nearly all the soreness which is so objectionable when rubber is used. With the use of the metal ligature, we hold the teeth firm as we move them, and make it possible to use the regulating appliance as a retainer, if we so desire.

I have mentioned a few points that are of vital importance to the man who endeavors to correct irregularities of the teeth, and have only treated them very superficially; but I hope that I have at least said something that will provoke some of you to give this subject more serious thought. Let us remember that this subject is not as difficult as we have been led to believe, but if we will only begin at the beginning and master our results, our success in the practice of orthodontia will be assured.—*Texas Dental Journal.*

